# Further explanations for the eq. (3) in “Estimating the daily trend in the size of the COVID-19 infected population in Wuhan”

**DOI:** 10.1186/s40249-020-00749-5

**Published:** 2020-09-15

**Authors:** Qiu-Shi Lin, Tao-Jun Hu, Xiao-Hua Zhou

**Affiliations:** 1 Beijing International Center for Mathematical Research, Peking University, Beijing, 100871 China; 2 School of Mathematical Sciences, Peking University, Beijing, 100871 China; 3 Department of Biostatistics, School of Public Health, Peking University, Beijing, 100191 China; 4 Center for Statistical Science, Peking University, Beijing, 100871 China

## Abstract

To avoid possible confusions to the readers, we provide further explanations for the eq. (3) in the research article “Estimating the daily trend in the size of the COVID-19 infected population in Wuhan” published in the *Infectious Diseases of Poverty*.

In our study [1], we proposed a new model to estimate the daily trend in the size of the COVID-19 infected population in Wuhan. When discussing this article with our colleagues, we found that a little more explanations for eq. (3) are needed to avoid possible confusions to the readers.

In the Methods section, we let *x*_*t*_ be the number of imported cases outside Hubei province on Day *t*, and derive a binomial model *x*_*t*_~*Binomial*(*N*_*t*_, *p*) if *t*_0_ + *d* < *t* ≤ *t*_0_ + 2*d*, and *x*_*t*_~*Binomial*(*N*_*t*_ − *N*_*t* − *d*_, *p*) if *t* > *t*_0_ + 2*d*. To make our model clearer, here *x*_*t*_ is in fact the number of infected individuals that travelled from Wuhan to places outside Hubei province on Day *t* − *d*. However, among the 10 940 confirmed cases in our data, 8546 (78.1%) do not have information on the date of departure from Wuhan, so the number of cases leaving Wuhan every day is unknown and hard to impute.

But the date of confirmation is available for each of the 10 940 cases, so we use the cumulative number of imported cases outside Hubei province as of Day *t* as approximation of ${X}_t=\sum \limits_{k=1}^t{x}_k$. Since the window from infection to detection is assumed as *d* days in our model, the infected cases that left Wuhan before Day *t* − *d* should be confirmed outside Hubei province before Day *t*, thus this approximation is reasonable and would not affect the conclusion in the paper.

## Data Availability

All data and materials used in this work were publicly available.
